# Gestion péri opératoire d'un paragangliome rétro-péritonéal

**DOI:** 10.11604/pamj.2015.21.300.6276

**Published:** 2015-08-26

**Authors:** Amine Bouslama, Jaballah Sakhri, Hamdi Echehoumi, Afraa Brahim, Fehmi Ferhi, Dhafer Ben Létaifa

**Affiliations:** 1 Service d'Anesthésie Réanimation CHU Farhat Hached, Sousse, Tunisie; 2 Unité de Recherche en Chirurgie UR: 12SP32, Service de Chirurgie Générale, CHU Farhat Hached, 4000 Sousse, Tunisie

**Keywords:** Paragangliome rétro-péritonéal, péri opératoire, tumeur endocrinienne, retroperitoneal paraganglioma, peri operative, endocrine tumor

## Abstract

Nous rapportons l'observation d'un patient âgé de 62 ans qui présente des douleurs abdominales avec une masse du flanc droit. Les explorations morphologiques ainsi que les données biologiques ont permis de faire le diagnostic d'un paragangliome secrétant. Les particularités diagnostiques ainsi que la gestion périopératoire sont envisagées.

## Introduction

Le paragangliome (PG) est une tumeur endocrinienne développée aux dépens des cellules chromaffines extra surrénaliennes. Les formes rétro péritonéales sont moins fréquentes que les autres localisations [1, 2]. Nous rapportons une observation d'un paragangliome rétro péritonéal avec des particularités révélatrices du diagnostic, et de prise en charge périopératoire.

## Patient et observation

**Figure 1:**
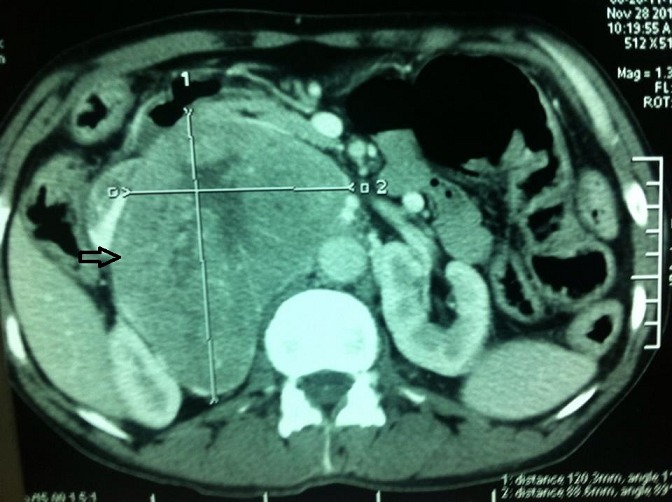
Flèche montrant une masse rétro péritonéale droite, prenant le contraste avec un centre nécrotique et mesurant 15 sur 12 centimètres

Un homme âgé de 62 ans avec une taille de 1.78 mètre et un poids de 86 kg (BMI: 27.1 kg/m2), diabétique depuis 3 ans et traité par Glimépéride à la dose de 8 mg par jour. Il a été hospitalisé pour des douleurs du flanc droit associées à une asthénie et une anorexie. L'examen clinique a trouvé une masse du flac droit fixe au plan profond de 12 cm de grand axe et pulsatile. Le reste de l'examen clinique était normal. Le patient avait une tension artérielle à 120 mm de mercure et une fréquence cardiaque de 70 battements par minute. Le scanner abdominopelvien, en mode hélicoïdal injecté, a montré une masse rétro péritonéale droite, prenant le contraste avec un centre nécrotique, à contours nets et mesurant 15x12×11 cm (Figure 1). Cette masse a un contact avec l'aorte sur une circonférence de 180°et responsable d'une compression de la veine cave inferieure. Une biopsie scannoguidée de la masse a été réalisée.

Au cours de cette ponction, le patient a présenté une poussée hypertensive, une tachycardie sinusale avec des sueurs profuses et des bouffées de chaleur faisant suspecter la nature sécrétante de la masse. L'examen anatomopathologique de la biopsie a conclu à un paragangliome. Un premier bilan de phéochromocytome était réalisé mettant en évidence des métanéphrines urinaires très élevées (normétanéphrine à 64mmol/24 heures, métanéphrine à 1,5mol/24 heures, somme normétanéphrine-métanéphrine à 65,5mol/24 heures, soit 21 fois la normale). La scintigraphie à la méta-iodo-benzyl guanidine (MIBG) a montré une intense fixation du tracé au niveau de la masse rétro péritonéale droite, sans autres foyers d'hyperfixation. Ainsi le diagnostic retenu était celui d'un paragangliome secrétant et fonctionnel. Ainsi un traitement chirurgical a été décidé. Dans le cadre de l’évaluation préopératoire une échocardiographie transthoracique a montré une fraction d’éjection ventriculaire gauche à 70%, des troubles de la relaxation myocardique avec un débit cardiaque conservé. Le patient a été mis sous Acébutolol 200 mg par jour et Nifédipine 20 mg par jour et ceci pendant 6 jours avant l'intervention.

Au bloc opératoire, le monitorage a comporté un électrocardioscope, mesure de la saturation pulsée en oxygène, mesure de la pression télé expiratoire de CO2, monitage de la température par sonde oesophagienne, mesure invasive de la pression artérielle par voie radiale, et mise en place d'une sonde vésicale. Une voie d'abord centrale, par voie jugulaire interne droite, a été posée. Une analgésie péridurale a été réalisée par insertion d'un cathéter péridural thoracique sous anesthésie locale au niveau T7-T8 L'induction anesthésique était réalisée avec: propofol 2 mg/kg et sufentanil 0,5 µg/kg intraveineux. L'intubation orotrachéale était facilitée par cisatracurium (0,15 mg/kg) et l'entretien était assuré par l'administration de sévoflurane et par des réinjections de sufentanil et de cisatracurium. La voie d'abord chirurgicale était une laparotomie sous-costale droite élargie vers la gauche. La technique chirurgicale comprenait une mobilisation prudente de la tumeur. Les pédicules péritumoraux étaient contrôlés en premier et ligaturées secondairement. Un tourniquet était mis sur la veine cave supra rénale à titre systématique. Le paragangliome est tracté par un fil et reséqué complètement. En peropératoire, les pics tensionnels définis par une pression artérielle supérieure à 160mm de mercure étaient contrôlés par de la nicardipine afin d'amener la pression artérielle systolique (PAS) entre 120 et 160mm de mercure. Cet objectif était obtenu à des doses variant entre 2 et 4 mg/h à la seringue électrique. Il n'y avait pas d’épisodes de tachycardie (supérieure à 100 battements par minute) tout le long de l'intervention. Après la résection tumorale, le patient a présenté une hypotension persistante malgré l'arrêt de la nicardipine et l'optimisation de la volémie par remplissage vasculaire nécessitant le recours à la noradrénaline à la dose 1 mg/heure. Les suites opératoires, pendant les premières 48 heures en réanimation chirurgicale, étaient marquées par une tendance à l'hypoglycémie corrigée par un apport calorique de sérum glucosé à 10%. L'analgésie péridurale a été poursuivie pendant 48 heures en postopératoire puis relayée par des antalgiques par voie orale. Un sevrage rapide de la noradrénaline était obtenu au bout de 24 heures. L'examen anatomopathologique de la pièce de résection a conclu à un aspect d'un paragangliome, avec une exérèse complète sans extension tumorale endovasculaire. Avec un recul de 18 mois, le patient demeure asymptomatique avec un scanner abdomino-pelvien normal.

## Discussion

Cette observation illustre les accès d'hypertension et de tachycardie au cours de la ponction de la masse rétro péritonéale, ce qui a fait fortement suspecté sa nature fonctionnelle. Initialement et avant la ponction scannoguidée, notre patient n'avait aucun signe évocateur de la sécrétion adrénergique de la masse rétro péritonéal. Le caractère sécrétoire a été démontré secondairement par le dosage des metanephrines. Les paragangliomes ou phéochromocytomes extrasurrénaliens sont des tumeurs développées aux dépens de cellules neuroectodermiques du système nerveux autonome [1]. La localisation retro péritonéale est rare et n'est retrouvée que dans 2% des cas [1, 2]. Sur le plan fonctionnel les céphalées, palpitations, sueurs abondantes sont retrouvée dans près de 90% des cas avec une hypertension artérielle [3, 4]. Une sténose digestive haute secondaire à un paragangliome rétro péritonéal, comprimant l′angle duodéno-jéjunal, a été rapporté [5].

Chez notre patient, seules les douleurs et la masse du flanc droit ont justifié un scanner abdominal et ce n'est que lors de la ponction de la tumeur qu'on a assisté à des pics hypertensifs et une tachycardie évoquant une sécrétion adrénergique. La mobilisation des cellules de la masse par l'aiguille aurait les mêmes conséquences physiopathologiques qu'une mobilisation peropératoire de la tumeur. Nous pensons qu'un holter tensionel de 24 heures, avant la réalisation de la ponction de cette masse retropéritonéale aurait détecter les variations tensionelles, ce qui aurait permis une préparation médicamenteuse et éviter les pics hypertensifs et la tachycardie, qui étaient de survenue brutale chez notre patient. D'ailleurs notre malade avait une fraction d’éjection ventriculaire gauche à 70%, des troubles de la relaxation myocardique à l’échographie ce qui témoigne de la présence d'une cardiopathie adrénergique secondaire à la sécrétion de la tumeur. L'hypertension artérielle peut être latente, paroxystique et des chiffres tensionnels normaux à l'admission n’éliminent pas le diagnostic, comme l'illustre notre observation. Nous pensons que le scanner associé à une ponction est suffisant pour faire le diagnostic, ce qui était le cas dans notre observation. l'IRM trouve son apport pour le diagnostic différentiel avec d'autres tumeurs et en cas de négativité des résultats de la biopsie [3, 6, 7]. La scintigraphie au MIBG a l'intérêt de détecter les localisations multiples et métastatiques. Sa négativité dans 10% des cas a été supplantée par d'autres explorations: l'octréoscan ou la tomographie par émission de positons utilisant divers traceurs. Ce bilan permet de planifier la stratégie thérapeutique ultérieure. La prise en charge d'un paragangliome nécessite une préparation préopératoire minutieuse pour éviter les complications cardiovasculaires pouvant survenir en per et postopératoire. Une résection chirurgicale complète constitue le seul garant de guérison avec des taux de survie de 75 et 45% respectivement à cinq et dix ans [1, 2]. Une évaluation de la fonction cardiaque par une échographie, s'avère nécessaire pour apprécier le retentissement de la sécrétion des catécholamines. L'effet de la préparation médicamenteuse préopératoire sur ces cardiopathies permet de fixer le moment optimum de la chirurgie. Cette préparation est basée sur le blocage des récepteurs alpha et béta adrénergiques pour minimiser les accès hypertensifs en péri opératoire ainsi que les troubles du rythme et de mieux gérer la volémie. La prescription des anxiolytiques permet de diminuer le stress pré opératoire. Dans notre observation, un traitement initial à base d'inhibiteurs calciques associé à un bêtabloquant ont permis de stabiliser la pression artérielle pendant une semaine avant l'intervention.

La voie d'abord chirurgicale, est classiquement une laparotomie médiane ou sous costale, mais actuellement des centres expérimentés ont rapporté des résections de paragangliomme, par voie laparoscopique [8, 9]. Le monitorage per opératoire comporte, en plus des éléments standards, la mesure de la pression artérielle par voie sanglante. Des moyens non ou moins invasifs tels que le monitorage par analyse de l'onde du pouls ou encore une échographie transœsophagienne, sont d'un grand apport pour détecter les variations des pressions de remplissage droite et gauche au cours des manipulations tumorales ou de l'expansion volémique rapide [10]. Nous pensons que l'analgésie péridurale permet de mieux contrôler les variations hémodynamiques peropératoires comme c’était le cas chez notre patient. L'anesthésie générale repose sur des produits non histaminolibérateurs. Une anesthésie profonde est indispensable. En raison des effets indirects sur la sécrétion des catécholamines, certains produits (kétamine, dropéridol, métoclopramide, tricycliques, phénothiazines) sont à éviter. Le propofol est l'agent d'induction de choix chez les patients présentant un état hémodynamique instable. L'entretien peut comporter de fortes doses de sufentanil et l'inhalation de sévoflurane en raison de sa rapide cinétique. La volémie doit être optimisée en continu. Les accès hypertensifs sont à craindre en peropératoire surtout lors de l'exérèse et la manipulation tumorale. Les arythmies et l'hypotension peuvent se déclencher après la résection du paraganglionome [10]. L'hypotension artérielle, voire le collapsus après ablation de la tumeur, doivent être prévenus par l'arrêt anticipé des vasodilatateurs et des bêtabloquants dès la ligature de la veine drainant la tumeur, et l'optimisation du remplissage associé à la noradrénaline, au mieux guidé par le monitorage hémodynamique [2, 6, 11].

En raison du sevrage brutal en catécholamines, les suites post opératoires peuvent être marquées par une instabilité hémodynamique pendant quelques jours [10]. Une surveillance de la glycémie est nécessaire en raison des hypoglycémies post opératoires. La prise en charge postopératoire de quelques heures, le plus souvent en salle de surveillance post-interventionnelle, est suffisante. Elle consiste en une surveillance hémodynamique, la détection de l'hypoglycémie et des complications chirurgicales.

## Conclusion

Les paragangliomes sont des tumeurs rares, longtemps asymptomatiques. L'exérèse chirurgical est le seul traitement radical, rendu plus efficace grâce aux progrès récents d'anesthésie réanimation par le contrôle des variations hémodynamiques induites par les catécholamines.
